# Cortical cells are altered by factors including bone morphogenetic protein released from a placental barrier model under altered oxygenation

**DOI:** 10.1042/NS20190148

**Published:** 2020-04-09

**Authors:** Veronica H.L. Leinster, Thomas J. Phillips, Nicola Jones, Sharon Sanderson, Katja Simon, Jon Hanley, Charles Patrick Case

**Affiliations:** 1 School of Clinical Sciences, University of Bristol, Southmead Hospital, Bristol, U.K.; 2 Dementia Research Institute, Cardiff University, Cardiff, U.K.; 3 School of Physiology, Pharmacology and Neuroscience, University of Bristol, Bristol, U.K.; 4 Translational Immunology Laboratory, NIHR BRC, John Radcliffe Hospital, Oxford, U.K.; 5 School of Biochemistry, University of Bristol, Bristol, U.K.

**Keywords:** Astrocytes, BeWo, Hypoxia, Neurodevelopment, Neurones, Rat

## Abstract

Episodes of hypoxia and hypoxia/reoxygenation during foetal development have been associated with increased risk of neurodevelopmental conditions presenting in later life.

The mechanism for this is not understood; however, several authors have suggested that the placenta plays an important role. Previously we found both placentas from a maternal hypoxia model and pre-eclamptic placentas from patients release factors lead to a loss of dendrite complexity in rodent neurons. Here to further explore the nature and origin of these secretions we exposed a simple *in vitro* model of the placental barrier, consisting of a barrier of human cytotrophoblasts, to hypoxia or hypoxia/reoxygenation. We then exposed cortical cultures from embryonic rat brains to the conditioned media (CM) from below these exposed barriers and examined changes in cell morphology, number, and receptor presentation.

The barriers released factors that reduced dendrite and astrocyte process lengths, decreased GABAB1 staining, and increased astrocyte number. The changes in astrocytes required the presence of neurons and were prevented by inhibition of the SMAD pathway and by neutralising Bone Morphogenetic Proteins (BMPs) 2/4. Barriers exposed to hypoxia/reoxygenation also released factors that reduced dendrite lengths but increased GABAB1 staining. Both oxygen changes caused barriers to release factors that decreased GluN1, GABAAα1 staining and increased GluN3a staining. We find that hypoxia in particular will elicit the release of factors that increase astrocyte number and decrease process length as well as causing changes in the intensity of glutamate and GABA receptor staining. There is some evidence that BMPs are released and contribute to these changes.

## Introduction

Abnormal blood flow in the placenta leading to acute hypoxia reperfusion has been linked to foetal brain damage [1]. This acute reperfusion can occur in a variety of conditions including miscarriage, pre-eclampsia, and intrauterine growth restriction [2].

One important clinical example is early onset pre-eclampsia where there is deficient conversion of the spiral arteries and damage to the myometrial segments, leading to an intermittent perfusion of the placenta and a low-grade ischaemia–reperfusion injury. Pre-eclampsia with an onset before 37 weeks is a significant risk factor for cerebral palsy. Some of the association is probably attributable to a high risk of pre-term birth that is also linked with early pre-eclampsia. However, a ‘direct’ effect of pre-eclampsia on foetal brain development also seems likely [3]. Children born to mothers with pre-eclampsia may present with cognitive defects [4,5] and they have a lower intelligence quotient at the age of 3 years [5]. The increased risk of intellectual disability is only partially explained by low birth weight [6]. Pre-eclampsia has also been linked with a greater risk of Attention Deficit Hyperactivity Disorder (ADHD) [3], and specific language impairment [7]. Any chronic placental insufficiency, which includes foetal hypoxaemia, can result in foetal growth restriction and long-term deficits in neural function including ADHD [8]. Recent imaging studies have revealed that adverse outcomes are strongly associated with reduced brain growth and neural complexity in later life. Increasing data suggest that these chronic deficits primarily reflect acute neuronal and glial injury sustained during adverse *in utero* events, such as exposure to severe hypoxia–ischaemia and inflammation [9]. While clinically many of these situations may present with chronic hypoxia in the placenta–foetal system, it is interesting also to consider how the placenta responds to hypoxia either in an acute event or before the onset of a chronic condition. Recently, there has been a particular interest in the contribution of placental pathology to neurodevelopmental disorders [10]. Furthermore, the placenta actively secretes molecules that are important for infant brain development and can be affected by gestational challenges [11,12].

Previously, we showed that the placenta or an *in vitro* model of the placental barrier responds to toxins or altered oxygen by secreting factors that cause genetic damage in fibroblasts or human embryonic stem cells [13,14].

We have also previously shown that the placenta will respond *in vitro* to hypoxia or hypoxia/reoxygenation by secreting factors that increase calcium and mitochondrial free radicals in embryonic cortical neurons *in vitro* and reduce synaptic activity, dendritic length, branching complexity, and spine density and the concentration of glutamate receptors [15]. These signals include altered levels of released BMP isoforms, changes in secreted microRNA and glutamate [17]. These changes include reduced BMP 4, 6, and 9 with increased BMP2 and another unidentified BMP isoform. We have also demonstrated that pre-eclamptic placenta explants *in vitro* also release secretions that similarly effect neuron dendrites and NMDA receptor concentration on cortical cells [16]. This work also demonstrated a possible second messenger system between neurons and astrocytes [16]. Under similar hypoxic conditions we demonstrated placental tissues experience oxidative stress [17]. Increased oxidative stress, mitochondrial dysfunction, and the production of reactive oxygen species (ROS) are well-known significant contributing factors to neonatal brain injury and neurodegenerative disease in later life [18]. Moreover, hypoxia, mitochondrial dysfunction, and ensuing ROS production may be a trigger of autophagy [19].

Our hypothesis is that molecules released from the placenta in response to the sudden altered oxygen levels could damage foetal white and grey matter *in utero* and provide a contributory cause for brain damage. In the present paper, we have used a simple *in vitro* model of the placenta, a bilayered barrier of BeWo trophoblast cells. We have explored whether hypoxia and hypoxia/reoxygenation of these barriers could induce them to release factors that damage cortical cells *in vitro* and what the nature of that signal may be. We have focussed on changes in astrocyte number and process length, dendrite lengths and glutamate and GABA receptors as these are known to be altered in the brains of patients with the psychological disorders that are linked with episodes of altered oxygen *in utero* [10,20–24].

## Materials and methods

### Cell culture

Mixed embryonic day (E) 18 rat cortical cultures were prepared as described previously [15] and were plated on poly-l-lysine (1 mg/ml Sigma) coated cover slips at a density of 10000 cells/ml and grown for 12 days before exposure to conditioned media (CM) for 6 days. Glial-only cultures were also developed from the same embryonic cells by being maintained with Dulbecco’s modified Eagle’s medium (DMEM; Sigma) 10% foetal bovine serum (FBS; Life Technologies). All cortical cultures are maintained in normal cell culture conditions (21% oxygen, 5% CO_2_, 37°C). All animal handling procedures were approved by the Animal Welfare and Ethical Review Body of the University of Bristol, and were conducted in accordance with the U.K. Animals (Scientific Procedures) Act 1986.

### Cell signalling inhibitors

Cell signalling inhibitors were applied directly to cortical cultures (1 h before exposure to CM) or indirectly via the *in vitro* model, the BeWo barriers (at exposure to altered oxygen) to see the effect on CM. Map kinase inhibitor SB203580 (20 μM; InviviGen), epidermal growth factor receptor (EGFR) inhibitor PD168393 (20 μM; Santa Cruz Biotechnology), Noggin (200 ng/ml; StraTech), Gremlin (Gremlin-2, 500 ng/ml; Sigma) and Chordin (chordin-like 1, 500 ng/ml; Sigma) were tested. Inhibitors were chosen to block signals in the Smad (Noggin, Chordin, and Gremlin), mTOr-Akt-PI3k (PD168393) and 38-MAPK (SB203580) pathways in an attempt to isolate which if any signalling pathway was being affected. Where appropriate Bone Morphogenetic Protein (BMP) binding antibody (R&D Mab3552) was applied to cultures at a dose of 10 µg/ml 1 h before exposure to CM.

### BeWo barriers and CM

Bilayered BeWo barriers were created as described previously [15]. In brief, BeWo human cytotrophoblast cells were seeded at 112000 cells/ml on to transwell inserts (Appleton Woods) and grown under standard conditions for 7 days until a confluent bilayer had formed. Barriers were then placed in the hypoxic chamber (Ruskinn Sci-tive) and exposed to 2% oxygen for 24 h. Medium below the barrier was collected as CM and frozen for exposure to primary cultures. For media which were conditioned by barriers exposed to 2–8, 2–12, and 2–21% oxygen, the barriers were exposed to 2% oxygen before increasing the oxygen for a further 24 h; in previous experiments the CM below BeWo barriers, which were exposed to hypoxia reoxygenation by increasing oxygen from 2% to either 8, 12, or 21%, all caused similar effects on dendrite lengths and Ca signalling in neurons, and on DNA damage in fibroblasts [15,17]. These have been proposed as physiologically relevant levels of hypoxia and re-oxygenation [25] in placental cells during pre-eclampsia. There are frequent difficulties associating atmospheric oxygen levels with partial pressures found in the blood. This was especially true when considering oxygen diffusion into culture media. In these models we have altered atmospheric oxygen rather than the level of dissolved oxygen in the media which may be considerably lower in accordance to gas diffusion laws. It is also important to note most labs use 21% oxygen as the condition to culture neurons despite it not being physiologically relevant. Notably, the effect on cortical cultures of CM from barrier cultured 21% oxygen and 8% oxygen were not shown to be significantly different from each other or the feeding media control [15,17]. With this in mind, it was decided to use 21% oxygen as a control in these experiments.

### Immunofluorescence

Cells were fixed with 4% paraformaldehyde (PFA; Sigma) and blocked with goat serum (Sigma) before incubating in primary antibody overnight at 4°C. Microtubule-associated protein 2 (MAP2; 1:2000, Synaptic Systems 188 004) was used to identify dendritesand glial fibrillary acidic protein (GFAP; 1:1000, Cell Signaling Ab 3670) was used to identify astrocytes. Previously in this model system all GFAP+ cells have been seen to be astrocytes. Cells were visualised using AF488 goat anti-guinea pig (Invitrogen A-11073) or AF546 donkey anti-mouse (Invitrogen A-10036) as well as DAPI (Vectorshield) to determine nuclei.

### Quantitative analysis

The images of immunoflourescently labelled cells on coverslips were captured (ISIS software) at 40× magnification on an Olympus BX41 microscope for quantitative analysis as described previously [15,17]. Briefly, a minimum of five images were taken per coverslip and saved as tiff files. The field of view was measured from calibrated scale as 72500 µm^2^. The images were then analysed using ImageJ software and the CellConter plugin to count cell types. Neurons and astrocytes were accepted as cells if they contained a defined nucleus based on DAPI staining. Cells were counted if the cell bodies were entirely in the field of view or only crossed the top or right border of the image. Data are expressed as cells per field of view. The averaged field of view (FOV) count was taken from the average of six independent replicates (from a different rat litter). Each independent replicate was the average of three coverslips plated with cells from the same rat brain dissociation. From each coverslip five images were taken as internal replicates. The cell density differences were taken to be not significantly different if the DAPI count was not significantly different between the internal replicates. The NeuronJ plugin was used to measure dendrite/process lengths by tracing the dendrites/process from the cell body and combining the total length per field of view (Supplementary Figure S1). For increased accuracy these measurements were repeated using Image Pro Premier software automate measurements which demonstrated non-significant difference to the NeuronJ measurement. These measurements were converted into µm with the use of a reference scale on a microscope slide (light microscope calibration standard).

### Glutamate and GABA receptor analysis

Slides from coverslips stained for GluN1, GluN3a, GABA Aα1, or GABA B1 were imaged using a confocal microscope (Leica AOBS SP2 x63). Seven images per cover slip were taken using Leica software. Quantification of these images was performed as previously decribed [15–17,26]. Briefly, ImageJ software was used to convert the images into RGB, and the intensity of fluorescence for each receptor stain was determined. This was then verified by cell counts based from DAP1 and levels of fluorescence in the absence of the cells and/or primary antibody to ascertain the background detection levels. Positive control was provided by measuring intensity of known concentrations of the secondary antibodies used. Microscope setting were maintained across all images. This was then verified by cell counts based from DAPI and levels of fluorescence in the absence of the neurons and/or primary antibody to ascertain the background detection levels. The background level of intensity was removed from the raw intensity to provide the true value [15–17,26]. The true value was then converted into a standardised level so that control values equalled to 1.

### Flow cytometry

Cells were plated at 700000 cells/T25 flask and grown for 12 days before exposure to CM for 24 h. Cells were detached using PBS EDTA and accutase. For flow cytometry there were 25000 cells per sample and for Image stream analysis there were 50000 cells per sample. Antibodies were used for the detection of astrocytes (GFAP 1:500 Millipore or GLAST 1:50 eBioscience), neurons (MAP2 1:2000 Synaptic systems 188 004 or CD90 1:200 eBioscience), autophagy (LC3 1:200 MBL and LysoID 1:500 Enzo Life Science) and a live/dead marker (1:2000, L23105 Life Technologies) and cell death (Annexin V 1:50, Life Technologies L23105). Mitochondrial function was accessed using Mitotracker Green FM 150 nM M7514, NaO 100 nM A1372, Mitosox 5 μM M36008 (all available from Life Technologies), and MitoID 1:2500 ENZ-51018, (Enzo Life Sciences). Cells were labelled for cell markers and mitochondrial functional indicators and the level of NaO, mitochondrial membrane potential, mitochondrial oxidative stress, and mitochondrial number. Cells from each exposure were compared for statistical difference from control. Flow cytometry was carried out on the BD LSRII and analysed using FloJo v 8.8.6. Cells prepared for detection of autophagy were run through Imagestream and analysed using IDEAS 6.1.303 software.

### Statistics

GraphPad Prism (version 5) or SPSS21.0 (IBM Corp., U.S.A.) were used to run statistical analysis on results. Data are presented as means ± SD and significance was set at *P*<0.05. Data were examined with Bartlett test and Kolmogorov–Smirnov test for variance and normality, respectively. As indicated in the figure legends, two-way or one-way ANOVA was performed with post hoc analysis using the Bonferroni correction. N (number) and *P*-value are indicated in the figure legends. N equals the number of separate times the BeWo cultures were grown and exposed to controlled oxygen levels. Each BeWo culture was used to make multiple barriers. Neurons/astrocytes were exposed to CM in three independent repeats. Each condition per repeat was performed in triplicate. Each different neuron/astrocyte culture that was grown was pooled from the offspring from different dams.

## Results

### Changes in morphology of neurons and GFAP^+^ cells

**Figure 1:**
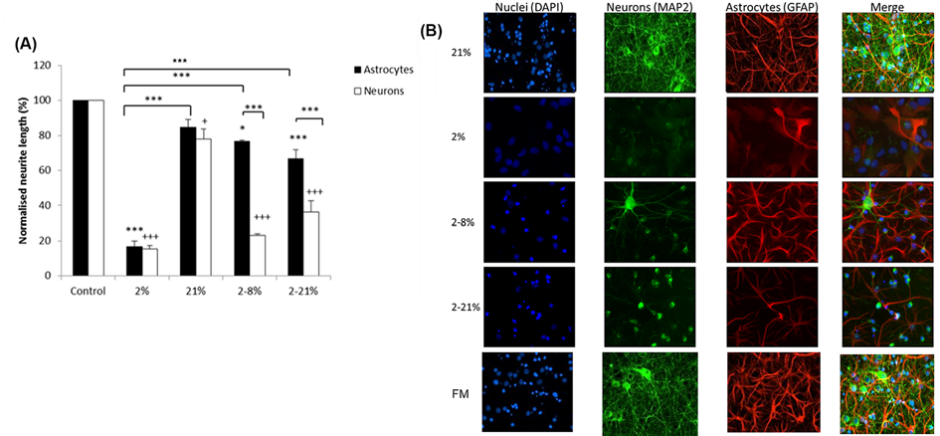
CM below barriers exposed to hypoxia, with or without reoxygenation, reduces neurite and astrocyte process length in cortical cultures (**A**) Mean (±SD) percentages of the process length of astrocytes and neurons after exposure for 6 days to the CM below BeWo barriers when they were compared with the length of GFAP^+^ cells and neurons that were exposed to normal culture medium (not below BeWo barriers). The latter lengths have been ‘normalised’ as 100%. The CM were taken below BeWo barriers that were grown at 21% oxygen or exposed for 24 h to a change of oxygen to 2% or from 2 to 8% or from 2 to 21%. *n*=3, Astrocytes: *P<0.05, ***P<0.001 when compared with control unless shown otherwise. Neurons: ^+^*P*<0.05, ^+++^*P*<0.001 compared with control. One-way ANOVA, post hoc Bonferroni (**B**). Examples of cortical cultures exposed to CM for 6 days. Images taken at ×20. Magnification scale bar = 70 μm.

Mixed cortical cultures (neurons and astrocytes), were exposed to media conditioned by the *in vitro* model, the BeWo trophoblast barriers, under different oxygen conditions (21, 2, 2–8, 2–21% oxygenation) and were stained for MAP2 and GFAP to identify the neuronal and glial (GFAP^+^ cells; in this model astrocytes) populations of the cultures, respectively. When exposed to media conditioned under control conditions (control media not below BeWo barriers and media below BeWo barriers at 21% oxygen) the cultures showed good neuronal growth and the GFAP^+^ cells had long processes with many branch points and end plates (Figure 1A,B). However, in cultures that were exposed to hypoxic CM there was a reduction in process length of both GFAP^+^ cells and neurons compared with control media below barriers at 21% oxygen (Figure 1). In contrast, media conditioned by trophoblast barriers under conditions of hypoxia reoxygenation caused a marked reduction of dendrite length but only a slight loss of GFAP^+^ cells process length (Figure 1).

**Figure 2:**
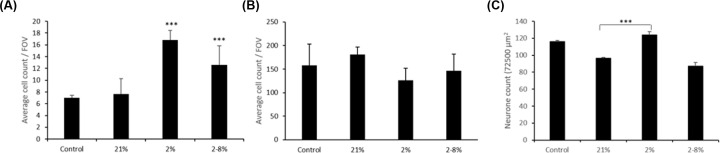
CM below barriers exposed to hypoxia, with or without reoxygenation, increases astrocyte counts in mixed (neuron and glia) cultures (**A**) Mean (±SD) number of GFAP^+^ cells per FOV at ×40 magnification in mixed cultures when exposed to the media below BeWo barriers which had been exposed to 21% oxygen or a change in oxygen down to 2% or from 2 to 8% as compared with control where cultures were exposed to normal media (control, not beneath BeWo barriers). *n*=6. One-way ANOVA, post hoc Bonferroni. *P<0.05, **P<0.01, ***P<0.001 compared with control. (**B**) Mean (±SD) number of GFAP^+^ cells/FOV ×40 magnification in glial only cultures when exposed to the CM described above. *n*=6. One-way ANOVA, post hoc Bonferroni. *P<0.05, **P<0.01, ***P<0.001 compared with control. (**C**) Mean (±SD) number of neurons/FOV in mixed cultures that were exposed to these CM ×40 magnification. *n*=6. One-way ANOVA, post hoc Bonferroni. ***P<0.001 compared with control.

This was not explained by a loss of the number of neurons or GFAP^+^ cells in culture (Figure 2A,C). Instead the number of GFAP^+^ cells per field of view (Figure 2A), and to a lesser extent of neurons (Figure 2C), was significantly increased after exposure to media conditioned by BeWo barriers under hypoxia (2%). There was a smaller increase in GFAP^+^ cells but not neurons when exposed to hypoxia reoxygenation (2–8%) CM (Figure 2A,C). The increase in GFAP^+^ cells was not seen when glial only cultures were exposed to the CM (Figure 2B). Also observed was a round soma in GFAP^+^ cells and increased blebbing (an indicator of dendritic retraction) in neuronal dendrites. Cell counts by DAPI stain correspond to changes in MAP2 and GFAP^+^ cell counts (Supplementary Figure S2).

### Autophagy and oxidative stress

**Figure 3:**
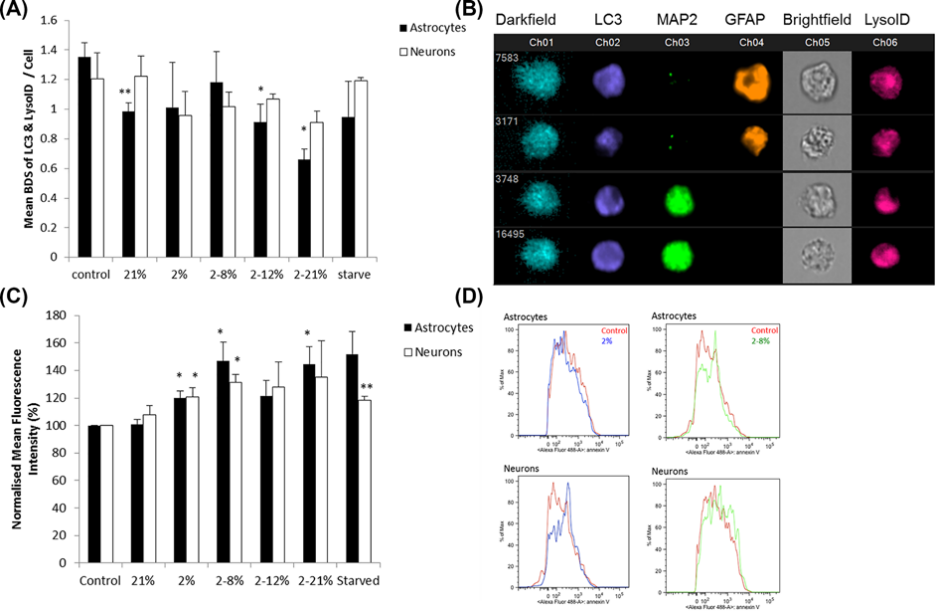
CM below barriers exposed to hypoxia, with or without reoxygenation, does not increase autophagy in mixed cultures (**A**) The mean (±SD) Bright Detail Similarity (BDS) of LC3 and LysoID in neurons and astrocytes as evaluated by ImageStream when mixed neuron and astrocyte cortical cultures were exposed for 1 day to the CM below BeWo barriers that were grown at 21% oxygen or exposed for 24 h to a change of oxygen to 2% or from 2 to 8% or from 2 to 21% as compared with cells which were exposed to normal tissue culture media (control, not conditioned below BeWo barriers). A positive control of starvation of cell cultures is also shown. *P<0.05, **P<0.01 when compared with control. Two-way ANOVA post hoc Bonferroni. *n*=6. (**B**) Examples of images of ImageStream analysis of Autophagy showing LC3 and LysoID co-localisation in neurons and astrocytes (**C**). The effects of hypoxic and re-oxygenation on cell death (Annexin V) in neurons and astrocytes, *n*=3. Two-way ANOVA *P*=0.0363. Student’s *t* test *P<0.05, **P<0.01 when compared with control of that cell type. (**D**) Representative flow histograms from neurons and astrocytes examined for Annexin V.

**Figure 4:**
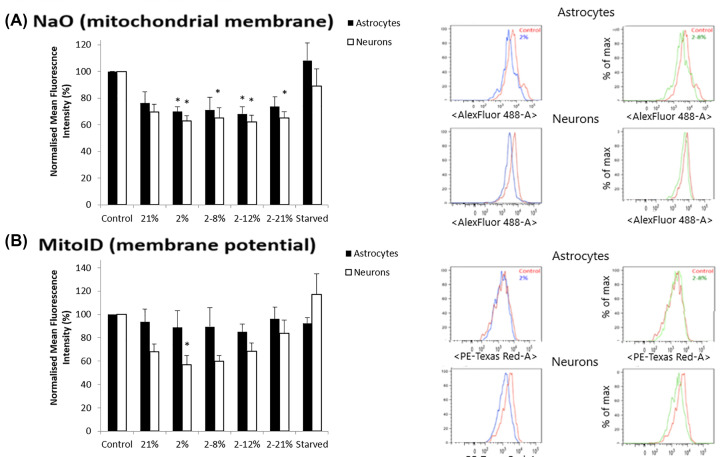
CM below barriers exposed to hypoxia, with or without reoxygenation, causes only minor changes in mitochondria in mixed cultures (**A**) Mean (±SD) fluorescent intensity of NaO staining, using flow cytometry, in neurons and astrocytes from mixed cortical cultures which were exposed for 1 day to the CM below BeWo barriers that were grown at 21% oxygen or exposed for 24 h to a change of oxygen to 2% or from 2 to 8% or from 2 to 21% as compared with cells which were exposed to normal tissue culture media (control, not conditioned below BeWo barriers). A positive control of starvation of cell cultures is also shown. *P<0.05 when compared with 21% condition of that cell type. Two-way ANOVA, post hoc Bonferroni. *n*=6. Flow cytometry graphs are shown in the right hand side. (**B**) Mean (±SD) fluorescent intensity of MitoID staining in the same neurons and astrocytes as in (A) using flow cytometry. *P<0.05 when compared with control. One-way ANOVA, post hoc Bonferroni. *n*=6. Flow cytometry graphs are shown in the right hand side.

Due to the dramatic loss of cell process in astrocytes and neurons, it was thought there would be an increase in autophagy to remove cellular debris from cultures. Surprisingly, there was a decrease in autophagy in GFAP^+^ cells under re-oxygenation (2–12%, *P*<0.05 and 2–21%, *P*<0.05, Figure 3A,B). There were subtle changes in cell death under hypoxic (2% oxygen, *P*<0.05, Figure 3C) and re-oxygenation (2–8% oxygen, *P*<0.05, Figure 3C) in both the neurons and GFAP^+^ cells, however, this does not explain the large changes in process length. There was only evidence of mild mitochondrial damage (Figure 4) in both GFAP^+^ cells and neurons where retraction of cell processes is observed. NaO (Figure 4A) showed a decrease in inner mitochondrial membrane health in hypoxic (*P*<0.05) for both GFAP^+^ cells and neurons and all other re-oxygenation conditions (*P*<0.05) for neurons but only 2–12% for GFAP^+^ cells. Significantly, the mitochondrial membrane potential (Figure 4B) was only decreased statistically in neurons exposed to 2% oxygen (*P*<0.05). Mitochondrial mass and superoxides were also measured but no significant changes with CM exposure were observed. The changes seen in cell death and mitochondrial damage do not reflect the large changes seen in morphology. Therefore, the next step was to explore any changes in glutamate receptor expression as well as the cell mechanisms involved. Damage to the astrocytes could lead to a reduced contact and support between neurons and astrocytes potentially inducing a more severe effect on the neurons as they are no longer being supported by the astrocytes.

### Receptors

**Figure 5:**
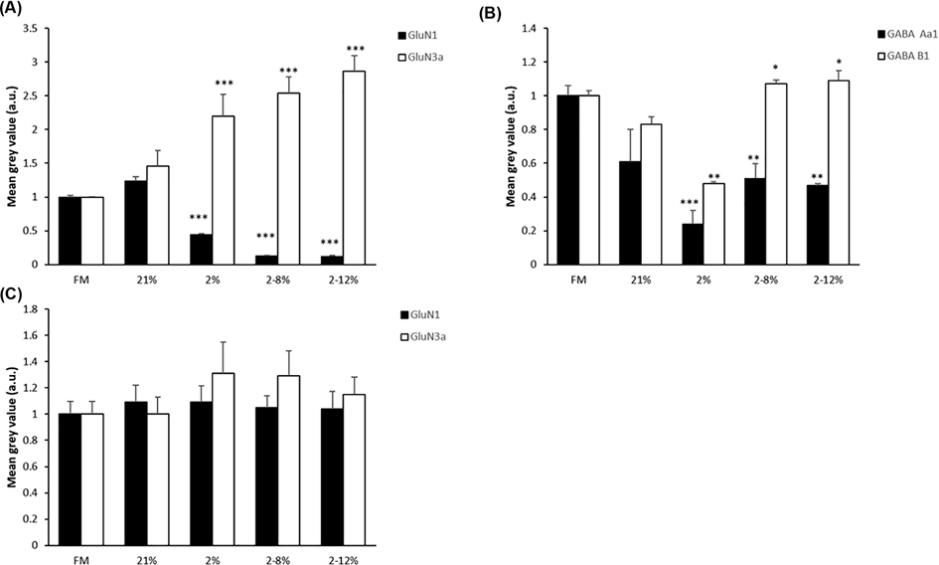
CM below barriers exposed to hypoxia, with or without reoxygenation, alters the intensity of staining of glutamate and GABA receptors in mixed cultures (**A**) Mean (±SD) intensity of GluN1 and GluN3 receptor subunit staining in mixed neuron and glial cell cultures when exposed for 6 days to media below BeWo barriers that had been grown at 21% oxygen or which had been exposed for 24 h to a change in oxygen to 2% from 2% to either 8% oxygen or 21% as compared with mixed cultures that had been grown in normal media only (FM = normal tissue culture medium not below barriers). *n*=9. One-way ANOVA, post hoc Bonferroni. *P<0.05, **P<0.01, ***P<0.001. (**B**) Mean (±SD) intensity of staining of GABA receptor subunit (GABA A α1 and GABA B1) detection in mixed neuron and glial only cultures which had been exposed to the same media as in (A). *n*=6. One-way ANOVA, post hoc Bonferroni. *P<0.05, **P<0.01, *** *P*<0.001. (**C**) Mean (±SD) intensity of GluN1 and GluN3 receptor subunit staining in glial only cultures which had been exposed to the same media as in (A). *n*=6. One-way ANOVA, post hoc Bonferroni. *P<0.05, **P<0.01, ***P<0.001.

The intensity of Glutamate and GABA receptors in culture were altered after exposure to media conditioned under altered oxygen (Figure 5) and similar to the changes described above there were differences in the response to media conditioned under hypoxia and hypoxia reoxygenation. The media conditioned by the *in vitro* model, the BeWo trophoblast barriers, under either hypoxia or hypoxia reoxygenation caused a decrease in GluN1 (Figure 5A) and GABA A α 1 (Figure 5B) staining and an increase in GluN3a staining (Figure 5A). However, the media conditioned by hypoxia caused a decrease in GABA B1 staining whilethat conditioned by hypoxia reoxygenation caused an increase in GABA B1 staining (Figure 5B). No changes were seen if glial only cultures were exposed to the CM (Figure 5C). It may be the effect in the mixed culture is mostly due to effects on the neurons rather than the glial cells.

These results suggest that although media conditioned under either oxygen change altered the lengths of cells processes and the intensity of receptor staining, the media conditioned by trophoblast barriers under hypoxia cause additional changes particularly to GFAP^+^ cells compared with media conditioned under hypoxia reoxygenation.

### Nature of the signal

**Figure 6:**
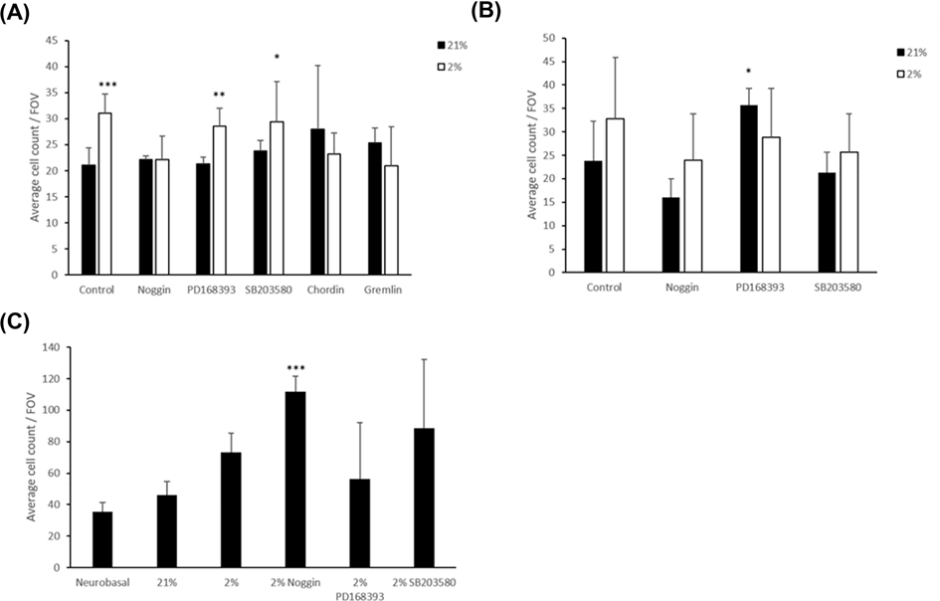
Effects of cell signalling inhibitors on the increased number of astrocytes after exposure to CM below barriers exposed to hypoxia (**A**) Mean (±SD) number of GFAP +ve astrocytes per FOV (×40 magnification) in mixed neuron and glial cell cultures that had been exposed to media below BeWo barriers that had been grown at 21% oxygen or had been exposed for 24 h to 2% oxygen. Values are shown after exposure to the CM with and without the presence of noggin, PD168393, SB203580, chordin or gremlin exposed to hypoxia through the BeWo barrier. One-way ANOVA, post hoc Bonferroni. *P<0.05, **P<0.01, ***P<0.001. (**B**) Mean (±SD) number of GFAP +ve astrocytes per field of view (×40 magnification) in glial only cultures which were exposed as described in (A). *n*=3. *P<0.05, **P<0.01, ***P<0.001, 2% compared with 21%. One-way ANOVA, post hoc Bonferroni. (**C**) Average number of GFAP^+^ cells per field of view (×40 magnification) in astrocyte only cultures. BeWo barriers exposed to inhibitors + hypoxia. *n*=3 ±SD. ***P<0.001 compared with 21% one-way ANOVA post hoc Bonferroni.

In view of the increase in GFAP^+^ cells number, the shortening of GFAP^+^ cells processes and a tendency, which was noted, for the cells to round up, it was of interest to test whether pathways that are associated with reactive astrocytes might have been activated after exposure to the media conditioned under hypoxia. To test this, we applied inhibitors of the SMAD pathway (noggin, chordin-like-1, Gremlin-2), mTOr-Akt-PI3k (SB203580) and P38-MAPK (SB203580) pathway to BeWo barriers, mixed cultures, and glial cultures. In mixed cultures and glial cultures neither the EGF receptor inhibitor PD168393 (*P*<0.01) nor the P38 MAPKinase inhibitor SB203580 (*P*<0.05) had an inhibitory effect on cell number (Figure 6A). Noggin (Smad inhibitor) reduced the total GFAP^+^ cells cell number to similar numbers recorded in control conditions (21% oxygen) in mixed cultures. In glial cultures, noggin, significantly reduced the number of astrocytes but not to the level recorded in the mixed cultures (Figure 6B). This indicates that the SMAD pathway is involved in the proliferation of GFAP^+^ cells and that neurons also have a role in astrocyte proliferation.

The effect of the antagonists on glial only cultures were tested indirectly by exposing the BeWo barriers to the antagonists before exposing them to the hypoxic CM 2% or control 21% which was then used as CM (Figure 6C). Two percent oxygen with Noggin CM caused a three-fold increase in cell number when compared with vehicle control which is in contrast with results observed from mixed cultures. This difference in effect of direct inhibitions on the astrocytes and indirect inhibition via the BeWo barriers shows that the BeWo barriers are releasing signalling molecules to the cell culture that cause proliferation of GFAP^+^ cells.

**Figure 7:**
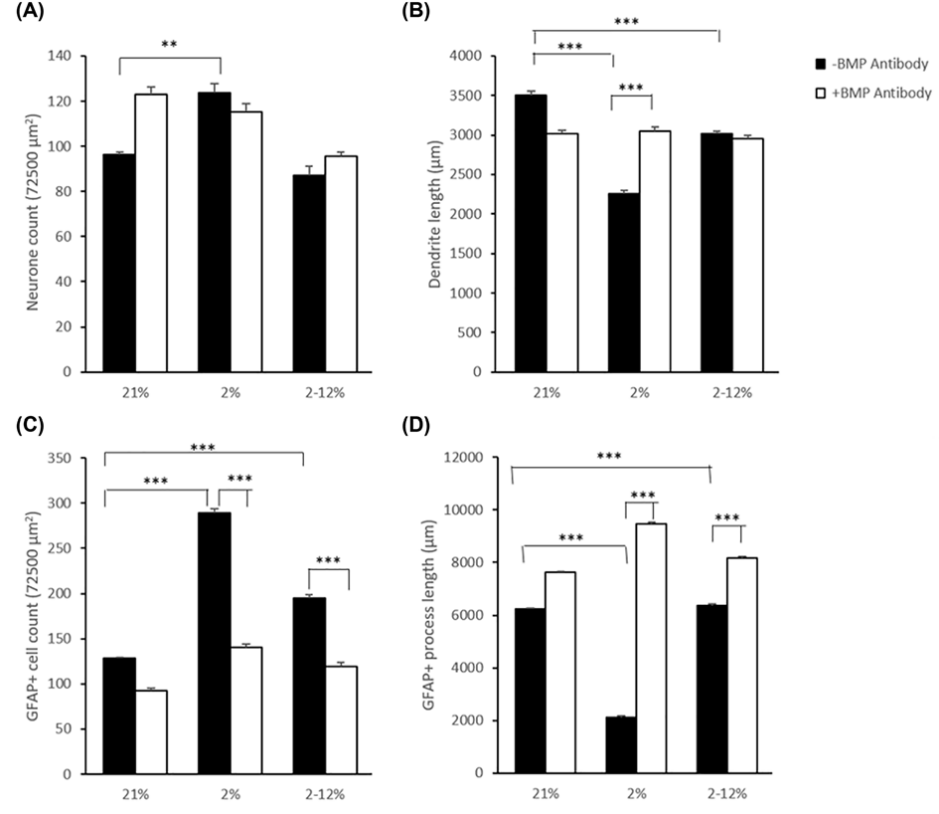
Effects of BMP binding antibody on mixed cultures exposed to CM (**A**) Mean (±SD) neuron count per FOV (×40 magnification) in mixed cultures that were exposed to normal culture media (control) or media below BeWo barriers that had been grown at 21% oxygen or exposed for 24 h to 2% oxygen or a change in oxygen from 2 to 12% for 24 h with (filled bars) or without (unfilled bars) BMP neutralising antibody. *n*=5. One-way ANOVA, post hoc Bonferroni. **P<0.01, ***P<0.001. (**B**) Mean (±SD) total dendrite lengths per FOV (×40 magnification) in mixed cultures exposed to the same CM as in (A). *n*=5. One-way ANOVA, post hoc Bonferroni. **P<0.01, ***P<0.001. (**C**) Mean (±SD) GFAP +ve cell count per FOV (×40 magnification) in mixed cultures exposed to the same CM as in (A). *n*=5. One-way ANOVA, post hoc Bonferroni. **P<0.01, ***P<0.001. (**D**) Mean (±SD) total GFAP +ve astrocyte process length per FOV (µm) in mixed cultures exposed to the same CM as in (A). **P<0.01, ***P<0.001, one-way ANOVA post hoc Bonferroni.

These results suggest the possibility that the barriers might be releasing members of the BMP family which are known to cause astrocyte proliferation and also to be inhibited by Noggin, Chordin, and Gremlin. In order to further confirm this possibility, we applied a neutralising antibody to the CM. Mixed cortical cultures incubated with a BMP binding antibody (BMP 2, 4) then exposed to hypoxia and hypoxia/reoxygenation CM. The antibody also provided partial protection (though not a full return to control) against dendrite reduction and neuron increase after exposure to media conditioned by hypoxia (Figure 7A,B). Cultures exposed with the neutralising antibody do not demonstrate the increase in GFAP^+^ cells counts seen after exposure to CM without the binding antibody (Figure 7C). The counts after exposures with the binding antibody were not significantly different from control but were significantly lower than the counts after exposure without the antibody (*P*≤0.001). The neutralising antibody also protected against the loss of GFAP^+^ process length after exposure to media conditioned by hypoxia or hypoxia/reoxygenation (Figure 7D). The protection was stronger in those exposed to CM from hypoxia however there was also an increase in those cultures exposed to media conditioned at 21% suggesting neutralising BMP causes a general increase in GFAP^+^ cells process length.

## Discussion

Altered oxygenation of the uterine environment during pregnancy is known to be a risk factor for damage to the foetal brain [22]. In addition, there is evidence that a change in oxygenation, even as early as the late first trimester, may increase the risk of a variety of psychological disorders in later life including schizophrenia, autism, and ADHD [10,20]. The mechanism for how this occurs is still not fully understood. Several authors have highlighted the importance of the placenta in this respect [10,23] including the possibility that the placenta may release factors in response to altered oxygen that could damage the brain [27]. Furthermore, it is known that the placenta actively secretes molecules that are important for infant brain development and which might be affected by gestational challenges [11,12].

In the present paper, we have used a simple *in vitro* model of the placenta, a bilayered BeWo trophoblast barrier to see if it would respond to altered oxygen by releasing factors that could cause changes in neurons and astrocytes in tissue culture. Previously we have demonstrated that CM from BeWo bilayered barriers produces a similar change in embryonic rat cortical neurons as CM from human placental explants [15]. We have also seen similar changes in cortical cultures after exposure to secretions from pre-eclamptic placenta explants [16]. We have also previously demonstrated that signals from placental cells are able to alter rat neurons and astrocytes during development in an animal model despite the presence of a blood–brain barrier [15,17]. Notably the blood–brain barrier has been shown to be more permeable the developing brain after ischaemia and reperfusion [30]. One drawback of this model is the CM from 21% oxygen exposed BeWo barriers does effect the cortical cultures slightly compared with feeding media controls. However the hypothesis we are presenting in this work is that there is significant difference between the effect of CM from barriers exposed to 21% oxygen and those exposed to hypoxia or hypoxia/reoxygenation.

The results show that both hypoxia and hypoxia reoxygenation of the *in vitro* model, a BeWo barrier, will cause it to release factors that result in a shortening of dendrites and GFAP^+^ cells processes and an increase in GFAP^+^ cells number. There was however a marked difference in the behaviour of GFAP^+^ cells according to the type of stimulus. Hypoxia of the BeWo trophoblast barrier caused it to release factors that caused a large decrease in the length of their processes and an increase in their number in culture in contrast with hypoxia reoxygenation which caused only slight changes in this regard. To what extent factors released by a placental trophoblast barrier *in vivo* during intrauterine altered oxygen may reach and damage a foetal brain is not certain.

As these cells were taken from E(18) rat brains there remains the possibility that the GFAP^+^ cells are not entirely astrocytes as radial glial cells can be GFAP^+^ at this developmental point. However in our previous experience of this model we do not find radial glial cells successfully surviving in these cultures. It is however worth considering the radial glial cells may play a role in the GFAP^+^ cell results.

In the present study, we examined changes to NMDA an GABA subunits these types of receptors are known to be changed in the brains of patients with neurodevelopmental disorders such as schizophrenia [28] and autism [29,30]. In tissue culture there was a loss of staining of the GluN1 receptor subunit which is the structural component of the NMDA receptor and an increased staining of the GluN3a subunit (inhibitory subunit which is present to a high degree in the neonatal brain [31]). This suggests a reduction in the number of functional NMDA receptors. This is a key change clinically in neurodevelopmental conditions [16]. The alteration of the GluN1 and GluN3a subunits have been found previously in both post-mortem and animal models of schizophrenia [28]. It has been proposed this change in NMDA receptor causes downstream effects in schizophrenia and is responsible for further changes [24,32]. Similarly in tissue culture there was a loss of staining of the GABAAα1 subunit which is found in the majority of GABA receptors and a variable change in GABA B staining depending on the nature of the oxygen change. A loss of GABA receptors is seen in brains of patients with autism [33,34] and there is also a specific loss of GABAAα1 subunit in layer 3 of the prefrontal cortex in schizophrenia patients [35]. Alterations in the GABA B receptors have been shown to be involved in the sleep dysfunction in schizophrenia [36]. This results in a heightened dopaminergic level in the brain.

In these tissue culture experiments, there was an increase in astrocytes’ numbers and a decrease in the length of their processes after exposure to hypoxia CM with only slight changes after hypoxia reoxygenation. Inhibitors of the Smad pathway including noggin which binds TGFβ proteins BMP 2, 4, and 7 preventing their access to their receptor, were able to prevent the increase in astrocytes in mixed cultures. Neutralising BMP 2/4 through the use of a BMP binding antibody also prevented the increase in astrocytes and prevented the shortening of the astrocyte processes. Interestingly it has been previously found that mesenchymal stem cells derived from human placental samples produce altered BMP2 levels when exposed to hypoxia [37]. This points to a possible change in release of a BMP from BeWo barriers after hypoxia to cause the reactive like changes in astrocytes. In keeping with this we found previously that there is a reduction in BMP 4, 6, and 9 and a pronounced increase in an unknown BMP in the media conditioned below BeWo barriers under hypoxia which was not so pronounced in media conditioned under hypoxia reoxygenation [17]. One possible future line of research could be to use RNA knockdown of specific BMP isoforms to further investigate the nature of this BMP signal.

It is possible that the difference in behaviour of astrocytes after exposure to CM from hypoxic and hypoxic re-oxygenated barriers might be explained by relative changes in these different types of BMPs in this conditioned medium as previously shown by Phillips et al. (2017) [17]. This concept would be in keeping with the findings that astrocytes *in vitro* respond directly to BMPs via phosphorylation of the Smad1/5/8 pathway. BMP signal promotes immature astrocytes to adopt multiple characteristics of mature astrocytes, including a more process-bearing morphology [38]. It induces astrocyte differentiation from oligodendrocyte precursor cells. BMPs are thought to play a key role in brain development [28] and cellular proliferation [30] *in vivo* under experimental conditions [39]. After chronic hypoperfusion of the brain, BMP4 promotes astrogliogenesis at the expense of oligodendrocyte precursor cell proliferation and maturation, thereby aggravating white matter damage [40]. In a clinical context, Chaboub and Deneen [41] and Edmonson et al. [42] have both drawn attention to the link between glial pathology and the autism spectrum disorders. There is increased expression of GFAP in the areas with disturbed neuronal architecture, therefore, suggesting an astrogliotic response with possible alterations in neurogenesis and neuronal migration [43,44]. It is therefore of interest that birth complications that are associated with trauma or ischaemia and hypoxia have also shown strong links to autism spectrum disorders, whereas other pregnancy-related factors without an ischaemic component such as maternal obesity, maternal diabetes, and caesarean section have shown a less strong (but significant) association with the risk of autism spectrum disorders [45,46]. This may suggest a possible partial interrelationship between hypoxia, BMP expression, and astrogliosis.

We have previously found additional factors, including glutamate [15] and microRNA [17], which have been released from the placenta *ex vivo* during oxidative stress. Some of these also alter the morphology of neurons *in vitro*.

There are several areas of further research that could be investigated in future studies. Further research into other signalling pathways such as other members of the TGFβ family. This would be particularly of interest if investigating any role or effect on microglia in the cell cultures. HIF1α is known to be up-regulated under hypoxia and has been found to be up-regulated in this model system [47]. Downstream of HIF1α it would be of interest to investigate erythropoietin and/or erythropoietin receptors in the model. As we have previously found microRNA [17] changes in placental secretions another possible line of investigation could be to perform RNAseq on the CM in combination with the BMP inhibition. Also of interested would be to investigate if particular neuron types are more effected than others or if the effect can be increased by the addition of more BMP to the media.

## Conclusion

In summary we have used a simple *in vitro* model of the placental barrier to test whether altered oxygen might induce it to release factors that cause changes to astrocyte and neurons in tissue culture. We find that hypoxia in particular will elicit the release of factors that increase astrocyte number and decrease process length. Whereas both hypoxia and hypoxia/reoxygenation changes in the intensity of glutamate and GABA receptor staining. There is some evidence that BMPs are released and contribute to these changes. Because changes in astrocytes are a particular feature in the brains of patients with autism spectrum disorders and in view of the association of these disorders with episodes of hypoxia during pregnancy it would be of interest in the future to test whether factors released by the placenta *in vivo* might include BMPs and might contribute to the aetiology of these disorders.

## Supplementary Material

Supplementary Figures S1 and S2

## Data Availability

All data generated or analysed during the present study are included in this published article (and its supplementary information files).

## Abbreviations

ADHD: attention deficit hyperactivity disorder
BMP: bone morphogenetic protein
CM: conditioned media
DAPI: 4′,6-diamidino-2-phenylindole
GABAAα1: Gamma-aminobutyric acid receptor subunit Aα1
GABAB1: Gamma-aminobutyric acid receptor subunit B1
GFAP: glial fibrillary acidic protein
HIF1α: Hypoxia-inducible factor 1-alpha
LC3: Light chain 3
MAP2: microtubule-associated protein 2
NMDA: N-methyl-D-aspartate
ROS: reactive oxygen species
TGFβ: Transforming growth factor beta 1
