# Supplementary material for: Cortical cells are altered by factors including bone morphogenetic protein released from a placental barrier model under altered oxygenation
Source: Neuronal Signal. 2020 Apr 9;4(1):NS20190148. doi: 10.1042/NS20190148 (PMC7363303; doi:10.1042/NS20190148)
Supplement: Supplementary Figures S1 and S2 [file NS-2019-0148_supp.pdf]

Supplementary figures: Cortical cells are altered by factors including bone morphogenetic protein released from a placental barrier model under altered oxygenation

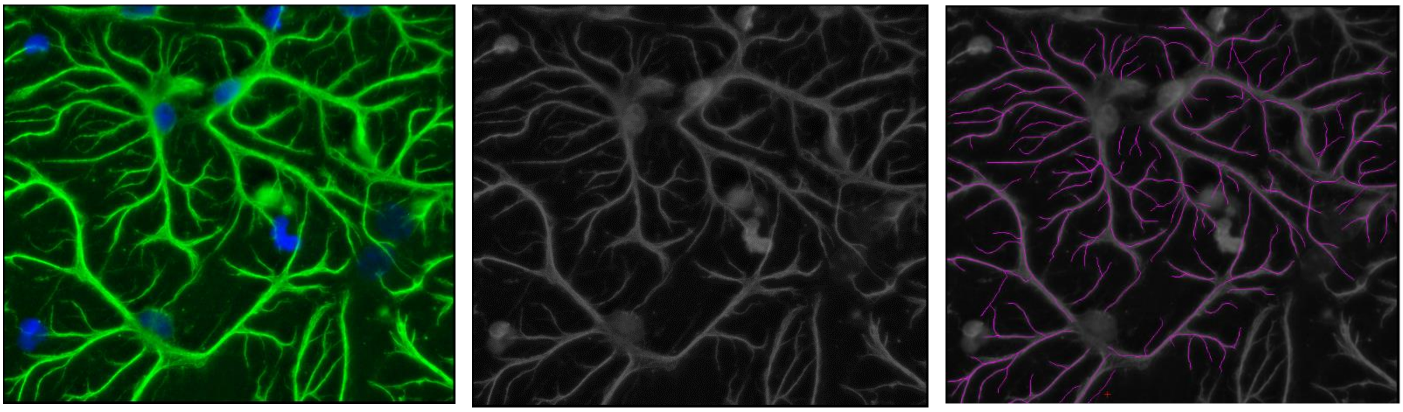

Supplementary Figure 1: *Measuring total dendrite/process length per field of view*

Sequential guide to measuring total astrocyte process length dendrite length (step A-C) using ImageJ plugin NeuronJ. This method was also used for measuring dendrite length using MAP2. A. Cortical culture immunostained with GFAP (astrocyte marker-green) and DAPI (nuclear marker-blue). B. The image was converted to a RGB 8 point Tiff using ImageJ then opened using the ImageJ plugin NeuronJ. C. DAPI and GFAP staining overlap was used to identify the cell body of the neurone and the tracing tool was used to trace process from the cell body (purple). Each process is traced from the cell bodies. Once all the process are traced NeuronJ is used to measure the total length of process in the image.

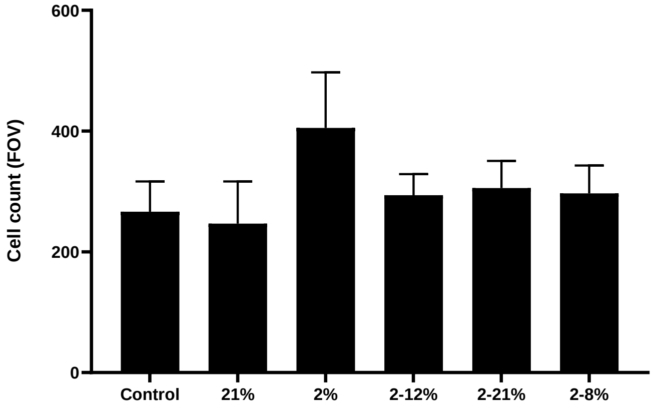

Supplementary Figure 2: *Cell count in mixed cultures*

Mean (±SEM) number of DAPI stained cells per field of view (FOV) at x40 magnification in mixed cultures when exposed to the media below BeWo barriers which had been exposed to

Supplementary figures: Cortical cells are altered by factors including bone morphogenetic protein released from a placental barrier model under altered oxygenation

21% oxygen or a change in oxygen down to 2% or from 2 to 8% as compared to control where cultures were exposed to normal media (control, not beneath BeWo barriers). N=6. One way ANOVA, post hoc Bonferroni. *=$p<0.05$, **=$p<0.01$, *** = $p<0.001$ compared to control
